# Astrocytes phenomics as new druggable targets in healthy aging and Alzheimer’s disease progression

**DOI:** 10.3389/fncel.2024.1512985

**Published:** 2025-01-06

**Authors:** Daniele Lana, Filippo Ugolini, Ludovica Iovino, Selene Attorre, Maria Grazia Giovannini

**Affiliations:** ^1^Section of Clinical Pharmacology and Oncology, Department of Health Sciences, University of Florence, Florence, Italy; ^2^Section of Pathological Anatomy, Department of Health Sciences, University of Florence, Florence, Italy; ^3^Institute of Neuroscience, National Research Council (CNR), Pisa, Italy

**Keywords:** hippocampus, astrocytes heterogeneity, clasmatondendosis, phagocytosis, beta-amyloid, neurovascular unit, syncytium, transcriptomics

## Abstract

For over a century after their discovery astrocytes were regarded merely as cells located among other brain cells to hold and give support to neurons. Astrocytes activation, “astrocytosis” or A1 functional state, was considered a detrimental mechanism against neuronal survival. Recently, the scientific view on astrocytes has changed. Accumulating evidence indicate that astrocytes are not homogeneous, but rather encompass heterogeneous subpopulations of cells that differ from each other in terms of transcriptomics, molecular signature, function and response in physiological and pathological conditions. In this review, we report and discuss the recent literature on the phenomic differences of astrocytes in health and their modifications in disease conditions, focusing mainly on the hippocampus, a region involved in learning and memory encoding, in the age-related memory impairments, and in Alzheimer’s disease (AD) dementia. The morphological and functional heterogeneity of astrocytes in different brain regions may be related to their different housekeeping functions. Astrocytes that express diverse transcriptomics and phenomics are present in strictly correlated brain regions and they are likely responsible for interactions essential for the formation of the specialized neural circuits that drive complex behaviors. In the contiguous and interconnected hippocampal areas CA1 and CA3, astrocytes show different, finely regulated, and region-specific heterogeneity. Heterogeneous astrocytes have specific activities in the healthy brain, and respond differently to physiological or pathological stimuli, such as inflammaging present in normal brain aging or beta-amyloid-dependent neuroinflammation typical of AD. To become reactive, astrocytes undergo transcriptional, functional, and morphological changes that transform them into cells with different properties and functions. Alterations of astrocytes affect the neurovascular unit, the blood–brain barrier and reverberate to other brain cell populations, favoring or dysregulating their activities. It will be of great interest to understand whether the differential phenomics of astrocytes in health and disease can explain the diverse vulnerability of the hippocampal areas to aging or to different damaging insults, in order to find new astrocyte-targeted therapies that might prevent or treat neurodegenerative disorders.

## 1 Introduction

After their discovery in the mid of the 19th century, for over a century astrocytes were considered “*that substance which lies between the proper nervous parts, holds them together and gives the whole its form in a greater or lesser degree*” (quoted from a Rudolf Virchow’s lecture, 3 April 1858). Astrocytes were subsequently described as cells that primarily provided support to neurons, and their activation, termed “astrocytosis,” was seen as a cellular reaction that set in motion mechanisms that were detrimental to neuronal survival. In the last 20 years, the scientific view on astrocytes has changed, and nowadays these cells are seen as fundamental protagonists in brain physiology.

Aside from protoplasmic astrocytes of the gray matter and fibrous astrocytes of the white matter, multiple types of specialized astrocytes are known to be present in the brain. Among them we can annoverate radial astrocytes, Müller cells of the retina and Bergmann cells of the cerebellum, velate astrocytes, surface-associated astrocytes, Gomori astrocytes of the arcuate, and pituicytes of the neurohypophysis (Verkhratsky and Nedergaard, 2018). We will focus this review on the heterogeneity of protoplasmic astrocytes of the gray matter, giving particular emphasis to hippocampal astrocytes.

Astrocytes are the most numerous and ubiquitous glia cells in the central nervous system (CNS) and have many housekeeping functions. Accordingly, they maintain CNS homeostasis and are responsible for neuroprotection and defense (Heneka et al., 2010; Sofroniew and Vinters, 2010; Allen and Eroglu, 2017; Verkhratsky and Nedergaard, 2018). Astrocytes have thousands of processes, which extend to the surrounding neuropil, define the space occupied by one single cell, are in touch with branches of neighboring astrocytes, but do not overlap with them (Kiyoshi and Zhou, 2019). In this way, astrocytes interact with other astrocytes to form a functional syncytium, which help their interplay with blood vessels, other glia cells, and neurons, to maintain the physiological functions of the healthy brain.

Astrocytes are an integral part of the blood–brain barrier (BBB), of the neurovascular unit (NVU), and of the glymphatic system, thus regulating neurovascular coupling, vascular tone, blood flow (Macvicar and Newman, 2015; Govindpani et al., 2019), and maintaining the influx of molecules that are used as trophic support for neurons and the efflux of waste or toxic molecules.

Astrocytes control the formation of neural circuits, regulate the development, maturation, and plasticity of synapses and release gliotransmitters necessary for synaptic plasticity (Lee et al., 2007; Perea and Araque, 2007; Verkhratsky et al., 2011; Navarrete et al., 2012; Araque et al., 2014). With all these mechanisms, astrocytes are involved in memory formation by mediating synaptic functions (Sofroniew and Vinters, 2010).

Protoplasmic astrocytes and fibrous astrocytes are classified according to their localization and structure. Fibrous astrocytes are localized in the white matter and support myelination processes, while protoplasmic astrocytes are located in the gray matter, have a bushy phenotype and directly contact blood vessels via their endfeet (Allen and Eroglu, 2017). However, it is becoming clear that this subdivision is rather too simplistic. Although evidence of substantial neuronal diversity between and within brain regions is now taken for granted (Saunders et al., 2018; Zeisel et al., 2018), the question of whether, similarly to neurons, different astrocyte subtypes exist in different brain regions and their exact role in physiological brain functions and/or pathogenic mechanisms remains quite unanswered. The difficulty to resolve this question mainly resides in the paucity of selective molecular tools and techniques that may help diversifying the populations of astrocytes *in situ*.

Astrocytes derive from progenitor cells that reside in the germinal zone during development, but their maturation evolves in a milieu that includes neurons, microglia, endothelial cells and oligodendrocytes. Thus, it is possible that the anatomical interplay between astrocytes and neurons during embryogenesis might sculpt and determine the differentiation and diversity of an astrocyte localized near a particular synapse, microcircuit or circuit. Interestingly, studies on neurons and astrocytes in coculture show that astrocytes promote neurite growth and synapse formation preferentially in cocultures derived from the same brain region. These data further reinforce the idea that the interaction between astrocytes and neurons drives astrocytes heterogeneity (Morel et al., 2017).

In the last 10 years several studies performed with different techniques have started to better define the phenomic of astrocytes, i.e., the complexity of their phenotypes and their related functions (Khakh and Sofroniew, 2015; Ben Haim and Rowitch, 2016; Khakh and Deneen, 2019). Chai et al. (2017), comparing hippocampal and striatal astrocytes, found significant differences in their Ca^2+^-sensitive K^+^ currents, suggesting strict regional specialization reflected in differential expression of genes encoding K^+^ channels. The striatum has many GABAergic neurons, while the hippocampus has primarily glutamatergic neurons. It appears, therefore, that striatal astrocytes have a lower requirement for K^+^ buffering and K^+^ dissipation. Indeed, hippocampal astrocytes have higher gap junction coupling and K^+^ currents than striatal astrocytes. Furthermore, striatal astrocytes branches cover larger territories, while hippocampal astrocytes have more interactions with neurons (Chai et al., 2017). This intra-regional morphological heterogeneity of astrocytes correlates with their physiological differential functions (Miller et al., 2019; Morel et al., 2019; Pestana et al., 2020). These differences are found in the cortex (Miller et al., 2019; Morel et al., 2019) and also in many other areas of the brain such as the brainstem, thalamus, cerebellum, and spinal cord. In these areas, astrocytes exhibit differential functional characteristics, such as the degree of synapse association (Chai et al., 2017; Lanjakornsiripan et al., 2018), Ca^2+^ signaling (Chai et al., 2017) and the ability to promote neuronal maturation (Morel et al., 2017). Astrocytes express many G protein-coupled receptors (Gi and Gq GPCR). Gq activation using a Gq-coupled Designer Receptors Exclusively Activated by Designer Drugs (DREADD) agonist elicits transient variations of intracellular Ca^2+^ and increase Ca^2+^-dependent gliotransmitter release from astrocytes, allowing a bidirectional communication with neurons (Van Den Herrewegen et al., 2021). Thus, activation of astrocytes, but not neurons, in hippocampal CA1, enhances memory acquisition in mice (Van Den Herrewegen et al., 2021). Specialized astrocyte subsets are responsible for the function of specific neuronal circuits, and are capable of synapse-specific regulation (Adamsky et al., 2018). Moreover, astrocytic Gi-DREADD activation is sufficient to elicit long-lasting synaptic potentiation in CA1 Schaffer collateral pathway in the absence of a high frequency stimulus (Van Den Herrewegen et al., 2021).

Furthermore, transcriptomic studies reveal that gene expression varies not only among astrocytes located in different brain areas, but also within the same brain region (Chai et al., 2017; Morel et al., 2017; Boisvert et al., 2018), providing evidence that astrocytes localized in different areas exhibit unique properties. Using single-cell RNA sequencing, five distinct astrocyte subtypes have been found in the mouse hippocampus, with distinct localization, different and specific morphologies, and differential intra-regional functions (Batiuk et al., 2020; Patani et al., 2023). Furthermore, it has been demonstrated that the morphology of each specific astrocyte subtype correlates with its brain localization (Batiuk et al., 2020; Patani et al., 2023).

Recently, Volterra’s group (de Ceglia et al., 2023) demonstrated in the hippocampus the existence of a subset of astrocytes that performs exocytotic release of glutamate following astrocyte-selective stimulations. Only peculiar astrocytes that have a defined anatomical localization within the hippocampus have exocytotic glutamatergic gliotransmission. This study adds a further level of complexity to the understanding of astrocytes phenomics (Batiuk et al., 2020; Bayraktar et al., 2020; Ohlig et al., 2021; Endo et al., 2022), and indicates that groups of specialized astrocytes, located in different areas, have diverse roles in physiological functions. The actions of these specialized astrocytes, including the enhancement of long-term potentiation (LTP) and memory, highlight their functional relevance (de Ceglia et al., 2023), despite their small numbers in the hippocampal astrocyte population.

The evaluation of astrocytes diversity and heterogeneity has been recently implemented by genetic sequencing based techniques such as droplet based single cell techniques (Saunders et al., 2018; Zeisel et al., 2018) and translating ribosome affinity purification (TRAP) (Doyle et al., 2008; Boisvert et al., 2018) which allow the comparison of transcriptomics of astrocytes in different brain regions. The output of these novel techniques is the demonstration that astrocytes are very diverse in different brain areas such as the striatum and hippocampus (Chai et al., 2017) and their transcriptome changes with age (Boisvert et al., 2018; Allen et al., 2023).

Differences in cortical layering of astrocytes, independent from neuronal layers, have also been demonstrated using large-scale single molecule fluorescence *in situ* hybridization (smFISH) (Smith et al., 2012). These cortical laminae of astrocytes are determined from their specific gene expression patterns such as *Chrdl1*, involved in synapse formation and maturation (Bayraktar et al., 2020). Similar results were obtained in the dentate gyrus of the hippocampus (Karpf et al., 2022) and in the dorso-ventral axis of the striatum (Chai et al., 2017). All these results indicate that the diversity of astrocytes might be more widespread and with higher physiological significance that previously thought.

Glial fibrillary acidic protein (GFAP) and S100β are still being used as markers in most studies on astrocytes (Ogata and Kosaka, 2002; Anlauf and Derouiche, 2013). Although still broadly in use, GFAP should be reconsidered as a solid and reliable astrocytic marker. Indeed, at least in mice, GFAP expression is often found to be affected by the age of the animals and the pathological context in which the analysis is performed (Middeldorp and Hol, 2011; Clairembault et al., 2014; Brenner and Messing, 2021). S100β also lacks specificity, being expressed in a subpopulation of mature oligodendrocytes, in some neurons and in epithelial cells of the choroid plexus (Rickmann and Wolff, 1995; Hachem et al., 2005), which makes the differentiation of specific astrocyte subsets rather difficult. Recently, other astrocytic markers have been identified by genetic profiling. Among them, the aldehyde dehydrogenase 1 family, member L1 (Aldh1L1) that is mainly present in cortical astrocytes (Waller et al., 2016), the excitatory amino acid transporter 2 (EAAT2), also known as glutamate transporter 1 (GLT-1), the excitatory amino acid transporter 1 (EAAT1) also known as glutamate aspartate transporter 1 (GLAST-1), and the glutamine synthetase (GS) (Williams et al., 2005). All these markers are less specific for astrocytes compared to GFAP, since GLT-1, GLAST-1, and GS are present also in neurons and oligodendrocytes (Schmitt et al., 2002). Nevertheless, at least over 90% of the expression of GLT-1 is astrocytic and GLAST is quite well accepted together with Aldh1L1 as a good astrocytic marker regardless of developmental stages (Mudannayake et al., 2016; Rimmele and Rosenberg, 2016; Pajarillo et al., 2019; Iovino et al., 2020).

Aquaporin 4 (AQP4), connexins 30 (Cx30) and Cx43 are mostly present in astrocytes endfeet, rather than in the cell soma (Nagelhus and Ottersen, 2013). Using combinatorial expression of astrocytes markers, it has been shown that astrocytes located in different brain regions express these markers in various combinations, further indicating the space-dependent differentiation of astrocytes (Khakh and Deneen, 2019).

The lack of univocal markers that identify phenomic astrocyte diversity *in vivo* is a challenge for the identification of intrinsic differences of astrocytes located in distinct brain regions. Nevertheless, the morphofunctional diversity of astrocytes that is starting to emerge from the recent scientific literature can explain the diverse functions that these cells have in the different brain areas. This insight could be crucial for understanding the regional susceptibility of the brain to insults or diseases, such as Alzheimer’s disease (AD), in which astrocytes are clearly implicated (Matias et al., 2019). Investigating the multiple phenomics and the contrasting roles of astrocytes in health and disease may unravel the pathogenetic mechanisms of many neurodegenerative disorders (Ben Haim et al., 2015). Although research on astrocytes has progressed more in the last few years than in the previous 100 years, many questions are still open, which the scientific research needs to answer to: what is the extent of heterogeneity of astrocytes and their physiological role within a particular brain region, such as the hippocampus? Can astrocytes heterogeneity determine the different susceptibility of different hippocampal areas to the same insult? Is disease progression dependent on this heterogeneity and, if it is, how do different astrocyte populations react to the disease in time and space? Do all astrocytes respond to injury and disease, or do different populations exhibit differential responses? What are the roles of reactive astrocytes in disease? Are they always toxic, or can they have beneficial effects depending on the astrocyte subset involved? Understanding astrocyte phenomics might also shed light on new pharmacological targets to find treatments that, modulating the reactivity of astrocytes, may control aging-dependent alterations and possibly neurodegeneration.

## 2 The phenomics of hippocampal astrocytes in aging

Aging of the brain is characterized by impairments of cognitive functions and by a variety of other neurobiological modifications and loss of function. In the Western countries, where the population life expectancy increases, aging is the main pathogenetic mechanism of AD and age-related cognitive decline represents a major challenge for the population at large, for the Health Systems, and for the scientific community. Indeed, much research is devoted to unravel the involvement of glia and neurons and their interplay in the mechanisms of aging, to find new treatments that may control the age-dependent brain alterations.

The modifications of organs, tissues and cells observed during the aging process are caused by a phenomenon known as “inflammaging,” the low-grade, chronic state of inflammation that develops over time with aging (Franceschi et al., 2007; Tennakoon et al., 2017). Inflammaging causes phenomic modifications and loss of function in all cells. In astrocytes, this phenomenon reduces their ability to maintain a physiological, healthy environment (Palmer and Ousman, 2018; López-Teros et al., 2022), altering their interrelationships with surrounding neurons, other glia cells, and endothelial cells of the BBB. However, it is not clear whether the diversity of astrocytes may have an impact on the way different brain regions age, and particularly the hippocampus, a region involved in the mechanisms of learning and memory.

**FIGURE 1:**
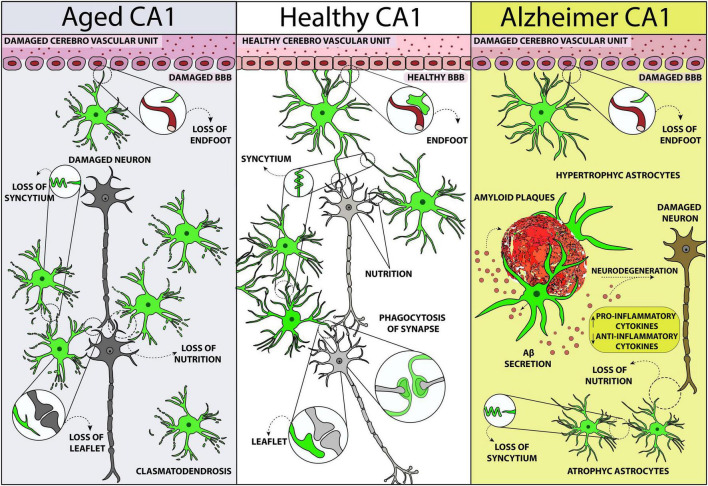
Schematic representation of astrocytes phenomic modifications in rodent CA1 hippocampus in healthy conditions **(central panel)**, in normal aging **(left)**, and Alzheimer’s disease **(right)**. The scheme is designed from the data obtained by Cerbai et al. (2012), Mercatelli et al. (2016), Lana et al. (2019) 2023, and Ugolini et al. (2018).

During aging, astrocytes located in the CA1 hippocampus of the rat change their phenomic, have shorter and twisted branches, lose their spatial orientation and become clasmatodendrotic (see Figure 1) (Penfield, 1928; Cerbai et al., 2012; Lana et al., 2016, 2019; Mercatelli et al., 2016; Perez-Nievas and Serrano-Pozo, 2018; Tachibana et al., 2019), consequently decreasing nutrients and oxygen delivery to neurons (Popov et al., 2023). Similarly, in the cortex of aged humans, astrocytes become atrophic, their branches become shorter, and their anatomical domains shrink. The branches of aged astrocytes have less gap junctions (Popov et al., 2023), with consequent interruption of their functional syncytium, as also shown in the rat hippocampus (Lana et al., 2019). However, as previously demonstrated in rats (Cerbai et al., 2012), astrocytes in the aged human brain show an upregulation of GFAP (Popov et al., 2023) but a negative regulation of ezrin, a protein localized in the leaflets, the fine distal and terminal astrocytic processes that make contact with synapses (Torres-Ceja and Olsen, 2022; Popov et al., 2023). Moreover, in the hippocampus of aged rats, GFAP expression increases with no proliferation of astrocytes (Long et al., 1998; Mouton et al., 2002; Cerbai et al., 2012; Lana et al., 2016). The increase of GFAP expression may be due to the elevated transcription of its soluble fraction in response to oxidative stress that characterizes the aging process (Sohal and Weindruch, 1996; Morgan et al., 1997, 1999; Wu et al., 2005; Middeldorp and Hol, 2011; Clarke et al., 2018). Since the increase of GFAP expression in the aged hippocampus is mainly due to the soluble form and not to the filamentous form, the observed discrepancy between GFAP levels and number of astrocytes may reside in differences of the proportion of soluble GFAP during aging (Iacono et al., 1995).

In the human brain, astrocytes make contact with their leaflets with up to 2 million synapses (Semyanov and Verkhratsky, 2021). In older adults, leaflets decrease in density, size, or both (Popov et al., 2023), reducing astrocytic interaction with synapses, limiting their homeostatic support, becoming less active in the elimination of excitatory synapses, facilitating the spillover of neurotransmitters, compromising neurotransmitter uptake and K^+^ clearance (Popov et al., 2021), and affecting synaptic plasticity (Valtcheva and Venance, 2019; Popov et al., 2021). All these age-dependent modifications recently demonstrated in the striatum of a Knock-in mouse model of Parkinson’s disease (PD) (Iovino et al., 2022) and in the human cortex (Popov et al., 2023), might well be present also in the hippocampus.

Vacuolization and swelling of the astrocytic cytoplasm, as well as disintegration and beading of their branches, are characteristics of clasmatodendrosis (Friede and van Houten, 1961; Tomimoto et al., 1997; Hulse et al., 2001), a response of astrocytes to energy failure and mitochondrial inhibition, which can cause dysfunction of the BBB (Friede and van Houten, 1961; Kraig and Chesler, 1990; Tomimoto et al., 1997; Hulse et al., 2001). Indeed, metabolic remodeling and increase of the oxidative metabolism (Yin et al., 2014) which limit the capacity of astrocytes to supply metabolic substrates to neurons (Jiang and Cadenas, 2014), as well as mild acidosis (Hulse et al., 2001), and Aβ deposition (Su and Chang, 2001; Sahlas et al., 2002; Ross et al., 2010), are all events that may cause clasmatodendrosis. The morphofunctional modifications of astrocytes caused by clasmatodendrosis, such as the shrunken arborization of their principal branches, are possibly responsible for the altered functionality of astrocytes that reverberates to other cells (Middeldorp and Hol, 2011). The shorter branches of clasmatodendrotic astrocytes may decrease the coverage of synapses (Rodríguez et al., 2009; Verkhratsky et al., 2010), causing an impairment in the support to synaptic transmission and possibly causing the progression to cognitive and psychiatric syndromes. Using astrocyte-specific CRISPR/Cas9-based gene knockdown of core genes in the hippocampus, a region where astrocytes exhibit high morphological complexity, Endo et al. (2022) discovered that reduction of *Fermt2* and *Ezr* proteins cause decreased astrocyte territory coverage (. In addition, parallel changes in cFos neuronal expression, and of pre- and post-synaptic markers, cause impairment in a cognitive task. These findings suggest that at least some phenomic changes of astrocytes may have causal effects on synaptic function, possibly contributing to disease phenotypes that may emerge in aging and AD (Endo et al., 2022).

However, even the general term “astrocyte process” that describes equivalently all the astrocytic branches is rapidly becoming too generic and sometimes misleading. Astrocytic processes are not identical, and a new nomenclature is needed. Astrocytes processes should be classified incrementally into branches, branchlets, and leaflets (Tong et al., 2013), according to the distance of the ramification from the soma. The increasing availability of astrocyte subcompartment markers will provide a more nuanced terminology to better describe those cellular subregions.

Furthermore, astrocytes have been shown to be phagocytic cells, and this activity is dependent on multiple EGF-like domains 10 (MEGF10) (Chung et al., 2013). The phagocytosis of excitatory and inhibitory synapses by astrocytes is fundamental for proper synaptic connectivity and plasticity in CA1 of adult mouse hippocampus (Lee et al., 2021). Astrocytes that lack the phagocytic receptor MEGF10 are less active in the elimination of excitatory synapses and cause the accumulation of functionally impaired synapses, defective long-term synaptic plasticity and impaired hippocampal memories (Lee et al., 2021). Astrocytes phagocytosis through MEGF10 is crucial for maintaining circuit connectivity and for supporting cognitive function. All these data contradict the previous notion that microglia are the sole mediators of synapse elimination (Paolicelli et al., 2011; Schafer et al., 2012). Indeed, it is emerging that astrocytes are involved in the recognition and clearance of obsolete or unwanted synapses via the atypical chemokine receptor 3 (Ackr3), a novel receptor that recognizes Cxcl12 bound to phosphatidylethanolamine at synaptic terminals (Giusti et al., 2024). However, during the aging process, astrocytes lose spatial orientation, coverage of synapses, and phagocytic activity of excitatory synapses, leading ultimately to impaired synaptic connectivity and neuronal homeostasis (Rodríguez et al., 2009; Verkhratsky et al., 2010). Astrocytes in the hippocampus of aged rats appear to lose their physiological functions, acquiring a further role in the disposal of neuronal debris (Cerbai et al., 2012; Lana et al., 2016).

The work by Bindocci et al. (2017) unravels a deeper level of astrocytes complexity in the hippocampus, demonstrating that single hippocampal astrocytes can have four different endfeet structures that interact with the vasculature. This morphological difference in astrocyte endfeet may represent a form of functional diversity (Bindocci et al., 2017), suggesting that the manner and the region in which different astrocyte processes are associated with the vasculature, or synapses, may be related to their different function. Endfeet modifications decrease the coverage of brain vessels compromising the BBB integrity (Chen et al., 2016), and the NVU. All these alterations, both quantitative and qualitative, can contribute to the modifications of the BBB and NVU, characteristic of aging and of the early stages of AD (Bell and Zlokovic, 2009).

The idea that in physiological conditions in the hippocampus there might be a heterogeneity of astrocytes populations in the different areas started since the paper by D’Ambrosio et al. (1998). They demonstrated that astrocytes in the Stratum Radiatum of CA1 and CA3 hippocampus have different electrophysiological properties (D’Ambrosio et al., 1998). The hippocampus is formed mainly by areas CA1, CA3, and dentate gyrus (DG), intercommunicating via the trisynaptic pathway (Basu and Siegelbaum, 2015). CA1 pyramidal neurons receive excitatory synaptic inputs from CA3 pyramidal neurons via the Schaffer collaterals or from the entorhinal cortex via the perforant pathway (Basu and Siegelbaum, 2015). The CA1 microcircuit is a major output of the hippocampus, fundamental for memory formation (Tonegawa and McHugh, 2008; Van Strien et al., 2009). Importantly, this activity, which is essential for the storage and retrieval of most hippocampus-dependent memories (Bartsch et al., 2010), is controlled by the synapse-interacting astrocytes. The highly ramified morphology of astrocytes allows them to maintain dynamic interactions with neurons, to modulate brain circuitries and behavior, to regulate homeostatic and synaptic mechanisms through close contact with NVU, glymphatic system, and extracellular matrix (Araque et al., 2014; de Oliveira Figueiredo et al., 2022; Hirrlinger and Nimmerjahn, 2022; Nagai et al., 2020; Oliveira et al., 2015; Oliveira and Araque, 2022; Rusakov et al., 2014; Viana et al., 2023). Recently, a paper published by St-Pierre et al. (2023), described ultrastructural markers of increased phagolysosomal activity in astrocytes throughout the hippocampal parenchyma of APP/PS1 mice. These astrocytes, named “dark astrocytes,” were found to be closely associated with the vasculature, and exhibited ultrastructural markers of cellular stress. Similar electron-dense, dark astrocytes were also found in an AD human post-mortem brain sample. This study provides the first thorough characterization of dark astrocytic state conserved from mouse to human hippocampus (St-Pierre et al., 2023).

The functional implications of astrocytes heterogeneity and the diverse electrophysiological responses in the contiguous and interconnected CA1 and CA3 hippocampal regions are still a matter of debate. Nevertheless, they may be implicated in synapse formation, maturation and maintenance, and thus in memory encoding. CA1 and CA3 differ in their vascularization since CA1 is less vascularized than CA3 from capillaries derived from the internal transverse artery (Coyle, 1978). Therefore, the wellbeing of neurons depends on the extent of astrocytes interconnections to form the functional syncytium in CA1 more than in CA3. Proper intercommunication of astrocytes with neurons is fundamental for the functional organization of the brain. Thus, the lack of integrity of the astrocyte syncytium that occurs during the aging process is responsible for the decreased oxygen and nutrient supply to the cells and has a negative impact on the survival of neurons in CA1 more than in CA3 (see also Zhou et al., 2024). Thus, the changes of cell communication networks that emerge with age or in a disease state, have important consequences especially in CA1 hippocampus, one of the brain regions more susceptible to insults.

For many years, astrocytosis (Nichols et al., 1993; Morgan et al., 1997, 1999), defined as significant increase of GFAP expression, has been the paradigm of astrocytic reactivity in most neurodegenerative disorders and aging. Nevertheless, in the last years the landscape is rapidly changing. Adaptive astrogliosis is demonstrated not to be always a negative phenomenon but, in some instances, it can be beneficial for neurons. In this respect, decreased activation of astrocytes may increase neuronal vulnerability, exacerbate the progression of pathological conditions, and impair tissue regeneration (Sofroniew, 2009; Burda and Sofroniew, 2014; Pekny et al., 2014). For instance, some data demonstrate that “astrogliosis” may sometimes reflect adaptive plasticity of astrocytes, as demonstrated in aged rodents in which an enriched environment increases their morphological complexity (Rodríguez et al., 2013; Sampedro-Piquero et al., 2014). Indeed, the view that astrocytes are merely latent toxic cells toward neurons is incorrect (Verkhratsky et al., 2023), while it is mainly the loss of supportive or protective functions from astrocytes that is noxious to neurons. Similarly, erroneous, incorrect, and misleading is the oversimplified idea that astrocytes polarize into simple opposing, A1 or A2 functional states, neurotoxic or neuroprotective, pro-inflammatory, or anti-inflammatory (Escartin et al., 2021; Paolicelli et al., 2022; Verkhratsky et al., 2023). Indeed, the so-called A1 and A2 astrocytes exhibit almost identical genetic profile and protein expression (Escartin et al., 2019; Clayton et al., 2024). On the contrary, it is starting to be understood that many different types of astrocytes exist, and most astrocytic responses are adaptive and allostatic, in favor of the recovery and regeneration of cells, rather than of their damage or destruction.

The activation profile of astrocytes can be considered as a continuum rather than an all-or-none phenomenon, and it is interesting to determine whether all astrocytes react to a similar stimulus/insult with the same phenomic modification, the so-called “astrocytosis” (Martín-López et al., 2013; Bribian et al., 2018), or whether they react in a more diverse and subtle way to a similar insult. The emerging idea that replaces the outdated concept of astrocytosis, or A1 subtype shift, is based on recent discoveries of the existence of different subtypes of astrocytes that probably react by setting up their own intrinsic responses, which may be diverse and independent from environmental stimuli and may vary during the aging process. Indeed, astrocytes reactivities to the same stimulus differ not only between astrocytes located in CA1 and CA3 hippocampus (Cerbai et al., 2012; Lana et al., 2016), but also within subregions of the same hippocampal area such as Stratum Pyramidalis and Stratum Radiatum (Lana et al., 2016). However, it is also possible that different signals derived from the environment cause diversification of the astrocytic responses (Martín-López et al., 2013; Bribian et al., 2018).

Astrocyte heterogeneity may even exist at the level of different terminal branches or leaflets of the same astrocyte that cover individual synapses, to finely tune synaptic transmission. Coherently, novel evidences are suggesting that astrocytes can create specialized synapses, which can drive complex behaviors (Holt, 2023). However, some key questions remain unanswered: how are the diversity and heterogeneity of individual astrocytes or branches established and maintained? Are they modified during development, adulthood or aging? What is their potential influence on aging and/or disease and injury?

## 3 The phenomics of hippocampal astrocytes in Alzheimer’s disease

As mentioned above, CA1 and CA3 hippocampal areas have critical, although different, roles in memory processing and develop significant functional, structural, and morphological alterations in AD (Bartsch and Wulff, 2015). Specific brain regions or group of cells are more vulnerable than others, and, indeed, AD pathology initiates in a region-specific manner. Selective vulnerability to neurodegenerative insults has been reported for CA1 hippocampal pyramidal neurons, both in experimental animal models and in humans (Mueller et al., 2010; Stranahan and Mattson, 2010; Small et al., 2011; Bartsch et al., 2015). Therefore, the comparison between these two hippocampal areas is of fundamental importance to enlighten the reason for these differences and possibly find new targeted therapeutic strategies.

In AD patients and in animal models of AD, alterations of astrocytes are highly heterogeneous in different brain regions, and can result in either hypertrophy or atrophy (see Figure 1) (Verkhratsky et al., 2010, 2015; Ugolini et al., 2018; Arranz and De Strooper, 2019; Lana et al., 2023). In the postmortem brain of AD patients, two different types of astrocytes, defined as A1 and A2 astrocytes with a now obsolete classification, are both present. They have a distinct phenotypic distribution in the different diseased brain areas (King et al., 2020), with a predominance of the neuroinflammatory and neurotoxic phenotype. Single-nucleus transcriptome analyses of the prefrontal cortex of AD patients have demonstrated the presence of transcriptionally diverse astrocytes, with three subpopulations that have disease-specific modifications of gene expression: downregulation of genes involved in synaptic signaling or upregulation of genes linked to cellular stress, and initiators of innate immune responses (Lau et al., 2020). Recently, Green et al. (2024) performed RNA-seq from more than 1.6 million nuclei isolated from dorsolateral prefrontal cortex of aged individuals to identify specific glia subpopulations associated with AD-related traits. The transcriptomes clustered into 16 microglia, 10 astrocytes, and 12 oligodendrocytes cellular subtypes. A reactive astrocyte state, Ast.10, characterized by expression of oxidative stress genes, was triggered by Mic.13 and tangles. The Ast.10 subtype should be explored to better understand how it is formed, how to prevent its formation, and whether its deactivation might be functional to personalized therapeutic treatments for AD prevention (Green et al., 2024). Furthermore, single-cell transcriptional analysis of the adult mouse nervous system revealed the existence of seven distinct astrocyte subpopulations which have regional defined distributions (Ståhlberg et al., 2011; Zeisel et al., 2015; Gokce et al., 2016). Such variety in rodents is overshadowed by the complexity of human astrocytes (Oberheim et al., 2009).

Recently, the role of GSH, one of the main endogenous antioxidant agents synthesized in the brain mainly by astrocytes and released through the ABCC1 transporter, has been evaluated (Ye et al., 2015). In transgenic mouse models of amyloidosis, the ABCC1 transport activity is increased to promote GSH release, possibly as a protective mechanism against oxidative stress, although this mechanism is not sustained in the long-term period (Zoufal et al., 2020). The Aβ isoforms Aβ_1–42_ and Aβ_25–35_ increase H_2_O_2_ production and GSH release in astrocytes (Allaman et al., 2010), and *in vitro* and *in vivo* studies have shown that monomeric forms of Aβ increase ABCC1 expression in acute and late stages of AD. Aβ is directly correlated with ROS production through the expression of inducible nitric oxide synthase (Chun and Lee, 2018) and can alter the antioxidant function of astrocytes mediated by GSH, although this may depend on the amyloid form, and the duration of exposure (Garg et al., 2011). The Aβ-induced process of astrocytic iNOS stimulation is dependent upon IL-1β and TNF, through NF-κB inducing kinase-dependent signaling (Akama and Van Eldik, 2000).

However, although it is evident that astrocytes can present gain- or loss-of-function in different areas of the AD brain, it is not clear whether these phenomic modifications are beneficial or damaging to the surrounding cells (Sofroniew, 2009; Pekny and Pekna, 2014). Apart from the protective roles brought about by astrocytes activation, such as production of anti-inflammatory factors, astrocytes can also acquire a toxic reactive phenotype, producing proinflammatory cytokines (Brambilla et al., 2009), increasing β-amyloid production (Nagele et al., 2003), or becoming atrophic and losing their neuroprotective functions (Diniz et al., 2017). Nevertheless, the matter is still controversial since the results are complex and sometimes contradictory. In mouse models of AD and multiple sclerosis (MS), it has been demonstrated that modulation of astrocyte reactivity improves functional deficits (Furman et al., 2012; Ceyzériat et al., 2018), accelerates plaques pathogenesis (Kraft et al., 2013), or causes no significant changes (Kamphuis et al., 2015; Colombo and Farina, 2016; Wheeler and Quintana, 2019). In addition, astrocytic intracellular pathways such as STAT3-dependent transcription are demonstrated to be beneficial in diseases such as traumatic brain injury (Levine et al., 2016), and in spinal cord (Anderson et al., 2016) and motor neurons injury (Tyzack et al., 2014), but detrimental in AD (Ceyzériat et al., 2018; Reichenbach et al., 2019). Therefore, STAT-3 and possibly other transcription pathways make astrocytes responses differ in different models of disorders.

In a 3xTg-AD mouse model, the PDAPP-J20 transgenic mice, and in adult mice intravenously injected with Aβ oligomers, astrocyte atrophy, characterized by reduced GFAP intensity, decrease in the number of astrocyte branches, and a reduction in the area covered by them, was observed in several brain regions, including the entorhinal cortex, medial prefrontal cortex, dentate gyrus, and hippocampal CA1, during the early stages of AD and along with disease progression (Olabarria et al., 2010; Kulijewicz-Nawrot et al., 2012; Beauquis et al., 2013). One of the possible functional consequences of astrocytes atrophy is the impairment of the BBB and NVU and decreased coverage of synapses, which may cause synaptic dysfunction and loss of metabolic support to neurons (Matias et al., 2019). Furthermore, Aβ-oligomers decrease astrocytes levels of TGF-β1, a cytokine that promotes the formation of synapses in the brain, further indicating new mechanisms involved in astrocytes-mediated synaptic dysfunction at the early stages of AD (Diniz et al., 2012, 2014; Araujo et al., 2016).

Dysbiosis, alterations of the gut microbiota, disorganize the colonic barrier and the BBB, allowing the passage from the periphery to the CNS of proinflammatory factors, immune cells and peptides such as Aβ, thus modifying the composition of the cerebral milieu and compromising the homeostasis of brain cells. Nevertheless, brain regions do not respond all in the same way to dysbiosis. For instance, in dysbiotic conditions caused by treatment with antibiotics, the expression of tight junction proteins decreases in the hippocampus, while it increases in the amygdala (Fröhlich et al., 2016), demonstrating a region-specific alteration of BBB permeability. These different conditions possibly increase the passage of damaging molecules only or preferentially to certain brain areas, with more intense damaging effects in the same regions.

As reported above, clasmatodendrosis changes astrocytes morphology, modifies their function (Jiang et al., 2018) and compromises the integrity of the BBB (Chen et al., 2016), of the NVU and of the glymphatic system. This phenomenon not only increases the passage of damaging molecules from the periphery to the CNS, but also reduces the disposal and clearance of interstitial Aβ and tau protein that can, in turn, accumulate in the brain parenchyma, implementing a vicious circle of neuroinflammation and tissue damage (Zhu et al., 2007; Miyazaki et al., 2011; Freeman and Keller, 2012; Guan et al., 2013; Wang et al., 2017; Sweeney et al., 2018). The dysfunction of BBB, NVU, and glymphatic system are involved in many neurodegenerative disorders, particularly those in which the accumulation of extracellular “waste” is of paramount importance in the pathogenetic mechanism. Particularly, clasmatodendrosis can hamper astrocyte-mediated Aβ clearance, decrease Aβ peptide disposal to the circulating system, and increase Aβ deposition in the brain parenchyma (Nagele et al., 2004; Marques et al., 2013; Mercatelli et al., 2016). In mouse models of AD, the impairment of Aβ clearance increases neuronal damage (Frenkel et al., 2013). Aging is the main risk factor for AD, and age-related changes in astrocyte function, further compromised by age-related dysbiosis, may be responsible for microlesions of the BBB, NVU, and glymphatic system, causing a reduction in Aβ peptide clearance and increasing the risk of amyloid plaque formation (Wang et al., 2017).

In the last years, it has been demonstrated that dysbiosis contributes to several neurodegenerative disorders such as AD (Mayer and Tillisch, 2011; Sharon et al., 2016; Cattaneo et al., 2017; Friedland and Chapman, 2017; Sun et al., 2020), PD (Hopfner et al., 2017), MS (Kadowaki and Quintana, 2020), and amyotrophic lateral sclerosis (Rowin et al., 2017). In a transgenic mouse model of AD, the APP/PS1 mice, the microbiota shows dysbiotic modifications (Traini et al., 2024) and treatments that recover the microbiota functionality shift a high proportion of astrocytes toward a protective phenotype (Lana et al., 2024). Especially in CA3 hippocampus astrocytes surround Aβ plaques and cooperate with microglia in the scavenging of Aβ plaques (Paolicelli et al., 2022; Lana et al., 2024), as also demonstrated in a different mouse model of AD (Ugolini et al., 2018). As pointed out above, the hippocampus, primarily affected in AD, is particularly susceptible to the products of the microbiota such as short chain fatty acids (SCFAs) (Sharon et al., 2016). Interestingly, decreased production of SCFAs (Unger et al., 2016) has been found in neurodegenerative diseases. Furthermore, immune cells expressing receptors for MB-deriving SCFAs can migrate to the brain through the BBB (Wang et al., 2018).

Recently, using snRNA-seq from 53 different AD brain tissue cohorts, Yu et al. (2024) identified in astrocytes a group of neurotoxic markers, ZEP36L, AEBP1, WWTR1, PHYHD1, DST, and RASL12, closely related to disease severity, and involved in inflammatory responses and in pathways related to neuron survival. In 5 × FAD mice, the marker WWTR1 is significantly increased in astrocytes that have elevated levels of GFAP (Yu et al., 2024). WWTR1 was thus identified as an important marker of inflammatory responses in neurotoxic astrocytes (Yu et al., 2024). WWTR1 is involved in the Hippo signaling pathway (Ray et al., 2022) and participates in cell proliferation, differentiation and tissue development (Fu et al., 2022). Dysregulation of Hippo signaling is associated to neurodegenerative disorders (Andl et al., 2017; Gogia et al., 2021). WWTR1, interacting and modulating NF-κB, a key pathway involved in inflammatory responses (Deng et al., 2018), may also modulate the astrocytic expression of pro-inflammatory cytokines, influencing the neurotoxic properties of astrocytes. However, further research is needed to fully elucidate the downstream WWTR1 mechanisms in astrocytes, whether these mechanisms are present in all astrocytes and their functional significance in AD. Recently, Dai et al. (2023) with snRNA-seq from normal, pathologic aging, and AD brains identified both increase of reactive genes and a marked decrease in homeostatic genes in protoplasmic astrocytes, correlated to amyloid pathology and loss of normal function. Upregulated genes were associated with cellular growth, responses to metal ions, inflammation, and proteostasis. Downregulated genes were involved in cellular interactions, neuronal development, ERBB signaling, and synapse regulation. Immunofluorescence staining confirmed downregulation of ERBB4 and transcription factor NFIA in reactive astrocytes (Dai et al., 2023).

The identification of markers of harmful astrocytes in AD is crucial not only to unravel the still unknown mechanisms of AD pathogenesis, but also to develop new targets of pharmacological intervention. It has been demonstrated that Aβ deposition causes hypertrophy of astrocytes especially in the CA1 hippocampus, and less in CA3 (Olabarria et al., 2010; Ugolini et al., 2018). Hypertrophic astrocytes are located in close proximity and surround Aβ plaques, both in animal models (Olabarria et al., 2010; Ugolini et al., 2018; Lana et al., 2024) and in the post mortem brain of AD patients (Meda et al., 2001; Mrak and Griffin, 2005). The localization of activated astrocytes around plaques is considered strategic and neuroprotective. In human AD patients, positron emission tomography (PET) shows that, at least in the prodromal stages of AD, the decrease in astrocyte reactivity parallels the ingravescence from mild cognitive impairment to AD, further demonstrating the neuroprotective role of astrogliosis.

Furthermore, in APP/PS1 mice it has been shown that astrocytes around Aβ plaques have upregulation of MAO-B which, together with the redistribution of the bestrophin 1 (Best1) channel (Park et al., 2009) may underlie the aberrant release of GABA and abnormal circuit firing observed in this model at early stages of Aβ deposition (Jo et al., 2014). More distantly from the plaques, astrocytes are atrophic. The underlying mechanisms of astrocyte atrophy as well as their functional impact on the onset of AD pathology have not been completely elucidated.

Although the contrast to the deposition of Aβ as a therapeutic strategy in AD is still debated (Karran and De Strooper, 2002), it has given important therapeutic outcomes that lead to the approval, although controversial, of aducanumab and lecanemab (see Chhabra et al., 2024). One hypothesis is that Aβ pathology drives tau pathology. Amyloid plaque need to be strongly reduced to reveal significant clinical benefit and the speed of amyloid removal appears to be fundamental for therapeutic benefits (Karran and De Strooper, 2002). Nonetheless, astrocytes have been demonstrated to be involved in AD pathogenesis as not only as a major source of Aβ in the neuroinflammatory context of AD, but also as major degradation station of Aβ via internalization of Aβ and enzymatic cleavage (Wyss-Coray et al., 2003; Montoliu-Gaya et al., 2017; Liu et al., 2017). On one side, stimulation of astrocytes with interferon gamma (IFN-γ) and TNF coincide with increased APP levels and beta-secretase-1 (BACE1) activation, with consequent increase in Aβ production (Heneka et al., 2005). Reactive astrocytes could be a major source of Aβ in the neuroinflammatory context of AD, but may also have neuroprotective effects at the early stages of amyloid production. This dichotomy could be due to heterogeneity of astrocytes and to the different microenvironment in which they are located.

The amplification of plaques deposition in mice models of AD by inhibition of astrocytes further stresses the neuroprotective role of astrocytes (Kraft et al., 2013).

Evidence of Aβ uptake, degradation and clearance by the then so-called reactive astrocytes was already demonstrated by Wyss-Coray et al. (2003). Phagocytosis of Aβ and secretion of Aβ-degrading enzymes by astrocytes near Aβ plaques (Yamaguchi et al., 1998; Kurt et al., 1999) may be regarded as the major mechanisms of astrocyte-dependent Aβ clearance (Perez-Nievas and Serrano-Pozo, 2018). Depletion of GFAP and vimentin increases the Aβ load in the APP/PS1 mouse model of AD, further demonstrating the protective role of astrocytes (Kraft et al., 2013). Astrocytes surround and infiltrates Aβ plaque to reduce neurotoxic Aβ species (Wyss-Coray et al., 2003; Xiao et al., 2014). Some astrocytes receptors such as LRP-1, low density lipoprotein receptor, and SRB1 (Garwood et al., 2011; Basak et al., 2012; Mulder et al., 2012) can mediate Aβ phagocytosis by astrocytes. Once in the cell, Aβ is degraded by enzymes such as insulin degrading enzyme, NEP, endothelin-converting enzyme-2 and matrix metalloproteinases (Xiao et al., 2014; Carter et al., 2019; Yin et al., 2006). Secreted enzymes such as α1-21 antichymotrypsin, α2-macroglobulin and apolipoprotein J help in Aβ catabolism in the parenchyma (Ries and Sastre, 2016; Carter et al., 2019), and anti-inflammatory cytokines such as tissue inhibitor of matrix metalloproteinase 1 (TIMP-1), soluble intercellular adhesion molecule 1 (sICAM-1), and transforming growth factor beta (TGFβ) influence plaque clearance in a rat model of AD (Chen et al., 2015; Saha et al., 2020). Furthermore, JAK-STAT seems the most important anti-inflammatory pathway regulating the function of astrocytes in several CNS insults (Okada et al., 2006; Herrmann et al., 2008; Wanner et al., 2013). Moreover, astrocytes secrete IL-6, IL-11, IL-19, IL-27, and sonic hedgehog that induce anti-inflammatory intracellular pathways and help in maintaining BBB integrity (Sarkar and Biswas, 2021). The chemokine CXCL8 can exert both protective and detrimental effects in the CNS (Mamik and Ghorpade, 2015), and the anti-inflammatory cytokines IL-6 and IL-11 have positive or negative effects (Sofroniew, 2015), depending on the downstream intracellular pathways they activate. The pro-inflammatory cytokine TNF-α can induce NF-κβ with proinflammatory effects or can upregulate A20, the ubiquitin-modifying protein that inhibits NF-κβ signaling (Catrysse et al., 2014), suppressing autoimmune inflammation (Wang et al., 2013). Furthermore, TNF-α, IFNγ, and IL-1β can induce galectin-9 expression that inhibits autoimmune inflammation (Steelman et al., 2013).

All these evidences add a further level of complexity to the spatio-temporal dynamics of astrocyte cytokines release. The same cytokine may have pro- or anti-inflammatory effects depending on the subtype of astrocytes and their localization and on the nature and progression of the neurodegenerative disorder. The identification of distinct astrocyte subtypes with their unique transcriptomic signatures and cytokine profiles will represent a major clue in the identification of pathogenetic mechanisms of neurodegenerative disorders and can become possible new therapeutic targets.

Endo et al. (2022) demonstrated that many known AD risk genes (e.g., Apoe, Clu, and Fermt2) are enriched in cortical and hippocampal astrocytes, brain areas primarily affected in AD. Using scRNA-seq on APP/PS1 mice, it was found that 11 disease expressed genes were upregulated and 629 were downregulated in comparison to controls. Several downregulated genes were correlated to astrocyte morphology and indicated that astrocyte territory size may be reduced in AD (Endo et al., 2022). Using scRNA-seq of APP/PS1 mice or snRNA-seq, it has been demonstrated that human AD genes related to astrocyte reactivity are not significantly altered, confirming previous work (Endo et al., 2022; Jiwaji et al., 2022). In the brain, astrocytes are the main producers of ApoE which, when secreted, has many effects. In particular, it has recently been shown in knock-in mice selectively expressing each of the human ApoE alleles, that ApoE4 expression impairs the formation of tight junctions and reduces the endfeet coverage of blood vessels, thereby compromising the integrity of the BBB. Conversely, removal of astrocytic ApoE4 production improves all of the above-mentioned phenotypes. Jackson et al. (2022) concluded that lowering the production of ApoE4 may be beneficial to maintain BBB integrity in subjects that carry one or two ApoE4 alleles, a population at higher risk to develop AD. The question this interesting paper does not address and which should be given an answer to is whether these effects of ApoE4 are due to modifications present in all brain astrocytes or only in a subtype of astrocytes localized in specific brain areas.

During the pre-clinical phase of AD, which begins decades before the clinical symptoms and continue during aging, extensive changes occur in glial cells and vasculature, which may orchestrate subsequent neuronal deficits. Soreq et al. (2017) found that age-related changes in astrocytes gene expression profile take place mainly in the hippocampus and to a lesser extent in other regions of the brain, further strengthening the involvement of astrocyte heterogeneity to the selective vulnerability in AD onset and progression. Nevertheless, a direct link between astrocytes phenomic changes with functional modifications in AD or other neurological disorders remains to be completely unraveled.

## 4 Possible therapeutic approaches targeting astrocytes in AD

The pharmacological treatment of most neurodegenerative disorders, among which AD, is still an unmet need. So far, the therapeutic strategies directed toward neurons, inflammatory mechanisms, or other non-cell specific treatments, have not given satisfactory results. The vision of neurological diseases as only neuronocentric should shift toward a wider view encompassing glia cells, particularly astrocytes, which play a role in the progression of several neurological conditions (Verkhratsky et al., 2017), and are becoming attractive targets for novel therapeutic strategies. Astrocyte-targeted therapies that reduce activation of astrocytes and the consequent inflammatory responses, that decrease astrocytes secretion of Aβ, or increase the production of protective factors, continue to emerge in the field of AD. The development of therapeutics that target astrocytes may have a broad range of applications. In various *in vitro* and *in vivo* models, it has been shown that these new approaches can slow the pathology of AD. In particular, improving subtype-specific beneficial roles, inhibiting subtype-specific detrimental roles or targeting subtype-specific cytokines may constitute novel therapeutic approaches to AD treatment. In addition, specific therapies that might uplift the beneficial role of subset of astrocytes, or that suppress the deleterious gain-of-function of astrocytes can be of great help in AD prevention or cure. Still, the field is in its infancy and much more of astrocytes diversity and function must be understood before proper therapeutical intervention could be considered for clinical use (for a thorough review on the current therapeutic approaches that act on astrocytes, see Rodríguez-Giraldo et al., 2022).

A continuous, auto-amplifying, positive feedback cycle of neuroinflammation/oxidative stress is present in the AD brain. Unfortunately, despite the potential of astrocyte-targeted therapeutic options, treatment with molecules that target pro-inflammatory mechanisms in astrocytes has produced limited, if any, clinical success, mainly because they are not specific and do not take into account the different involvement of astrocytes in different phases of AD progression. Many compounds with antioxidant potential which target astrocytes have been proposed. Among them, phloroglucinol (Yang et al., 2021), nobiletin (Wang et al., 2022), curcumin (Yu et al., 2022; Daverey and Agrawal, 2016), and many others (for an extended review, see Rodríguez-Giraldo et al., 2022). Although these compounds may be promising, the possibility of using them in therapy is still far.

Among many different proposed new treatments currently under scrutiny, the agonists of the glucagon-like peptide-1 receptor (GLP-1RA), approved for type 2 diabetes mellitus (Habib et al., 2020) appear promising. GLP-1 receptor is expressed in the brain, in areas involved in learning and memory (Park et al., 2021). GLP-1RAs protect astrocytes *in vitro* and improve cognitive dysfunction *in vivo* (Xie et al., 2021; Zhang et al., 2022). A long lasting GLP-1R agonist, NLY01, a brain-penetrant pegylated analog of exenatide, seems to block neurotoxic astrocytes (Sterling et al., 2020; Gharagozloo et al., 2021; Park et al., 2021).

At present, one of the most promising target of therapeutic intervention is TNFα, since the observation that rheumatoid arthritis patients treated with the TNFα inhibitor Etanercept have lower risk to develop AD (Chou et al., 2016). Nevertheless, in phase-2 clinical trials the drug did not give conclusive outcomes (Decourt et al., 2016). These negative results could be due to the contrasting effects that anti-TNFα treatment can have on astrocytes in the advanced stages of the disease, inhibiting their toxic activation, or in the prodromal phases, triggering the beneficial intracellular signaling factor A20 (Sofroniew, 2015). Nevertheless, many currently ongoing clinical trials are focused on inhibition of Pioglitazone, an agonist of peroxisome-proliferator-activated receptor gamma (PPARγ), acts on astrocytes and regulates metabolic coupling of astrocytes/neurons (Cowley et al., 2012), promotes the formation of dendritic spines and synapses (Moosecker et al., 2019), ameliorates amyloid and tau pathology in animal models (Singh, 2022), and improve learning and memory (Cowley et al., 2012). Unfortunately, despite promising preclinical data, the drug showed no clinical efficacy (Zhang et al., 2024).

Novel approaches in AD treatment are the use of beneficial cytokines. Administration of tissue inhibitors of metalloproteinase-1 (TIMP-1), a cytokine produced by protective astrocytes, increases Aβ disposal, inhibits neuronal apoptosis, improves synaptic health and ameliorates cognitive deficits in a rat model of AD (Saha et al., 2020).

Furthermore, TGFβ and IFNβ administration improve memory impairments, neuronal apoptosis and synaptic plasticity in mouse and rat AD models (Chen et al., 2015; Chavoshinezhad et al., 2019; Hu et al., 2019). These and other strategies such as the use of IL-33 to APP/PS1 mice with improvement of cognitive functions (Fu et al., 2016), although promising, require further investigation.

Other strategies to enhance the neuroprotective actions of astrocytes are currently being investigated. Among these, the use of the calcilytic molecule NPS 2143 has been proved reduce the excessive secretion of Aβ42 and Aβ load (Armato et al., 2013). Furthermore, NaBP (Na sodium phenylbutyrate), a drug used to treat urea cycle disorder, promotes the secretion of BDNF and NT-3 by astrocytes via CREB activation in a 5 x FAD mouse model (Corbett et al., 2013).

Gene editing has been used to explore astrocyte-targeted therapy for AD such as the induction of NRF2 using a lentivirus NRF2 vector with reduction of Aβ secretion by astrocytes, normalizes cytokine release, and increases GSH secretion in human Presenilin-1 mutated astrocytes (Oksanen et al., 2020). Nevertheless, astrocyte-specific delivery vectors either with adenovirus or lentivirus are currently being studied, the actual clinical-grade vectors display limited cell-type specificity and non-optimal biodistribution. Hence, their development is still a process that needs to be refined.

Another interesting method is the delivery of siRNA to astrocytes, by coupling chitosan nanoparticles (NPs) to transferrin receptor and bradykinin B2 receptor antibodies, and exploiting the transcytosis machinery of the BBB (Gu et al., 2017). Upon intracerebroventricular administration, lipid NPs functionalized with apolipoprotein E have been used to deliver mRNA to astrocytes and neurons to mice to increase protein expression (Tanaka et al., 2018).

Furthermore, microRNA-592 (miR-592), may play a role, since its downregulation *in vivo* inhibits astrocytes injury caused by oxidative stress, and increases *in vitro* their viability (Wu et al., 2020). These exciting new approaches toward protective astrocytes provide new insights into new therapies for AD.

More research is needed to assess the efficacy and safety of new therapeutic strategies that target astrocytes in the treatment of AD. Furthermore, understanding the involvement of other factors in neurodegeneration and the complex interplay between astrocytes, microglia, and neurons is critical to find effective treatments for AD. Until preventive methods/drugs are not available, approaches to block the progression of neurodegeneration and to promote the correct structural and functional recovery of damaged neural circuitries are needed for an efficient treatment against AD. Aside from the approved therapeutic strategies already in the clinic, many other compounds have shown potential efficacy toward AD in preclinical studies, however, since they do not target specifically astrocytes, they have not been included in this review.

## 5 Conclusion

Accumulating evidence show that astrocytes are heterogeneous subpopulations of cells that differ from each other in terms of transcriptomics, molecular signature, function, and response in physiology and pathology and are critically importantly for formation and function of the healthy CNS. Heterogeneity is diffused not only among, but also within different brain regions and it is likely responsible for interactions essential for the formation of the specialized neural circuits that drive complex behaviors. New tools and experimental strategies are needed to understand the exact range of astrocyte heterogeneity *in vivo* and its functional consequences. In AD, according to their spatial location, astrocytes modify their phenomics and functions not only in a diverse way close or far from Aβ plaques, but also differently in different brain areas. Astrocyte reactivity is not just a hallmark of aging and brain diseases, but represents a key mechanism involved in the pathogenesis and progression of these conditions. From all the above, it is evident that the knowledge on astrocytes is evolving very fast, but much more research is needed to understand their exact role in brain physiology and pathology to find possible therapeutic targets for the development of effective drugs. As suggested recently by Green et al. (2024), averting the polarization of astrocytes into specific phenomics such as Ast.10 opens new avenues for therapeutic interventions that might prevent the manifestations of AD. Failure to understand astrocytes heterogeneity and to modify their responses in pathological conditions represent currently undervalued concepts in the development of novel therapeutic strategies and may explain the continued failure of CNS drugs to have therapeutic efficacy.
